# Novel Predictors of Preeclampsia and Pregnancy Complications: the Impact of Type 1 Diabetes Mellitus on Maternal and Fetal Circulatory Levels

**DOI:** 10.33549/physiolres.935424

**Published:** 2025-06-01

**Authors:** Anna CINKAJZLOVÁ, Kateřina ANDERLOVÁ, Markéta HORNOVÁ, Antonín PAŘÍZEK, Miloš MRÁZ, Michal KRŠEK, Martin HALUZÍK, Patrik ŠIMJÁK

**Affiliations:** 1 Institute of Medical Biochemistry and Laboratory Diagnostics, First Faculty of Medicine, Charles University and General University Hospital, Prague, Czech Republic; 2 Centre for Experimental Medicine, Institute for Clinical and Experimental Medicine, Prague, Czech Republic; 3 Clinic of Gynecology, Obstetrics and Neonatology, First Faculty of Medicine, Charles University and General University Hospital, Prague, Czech Republic; 4 Third Department of Medicine – Department of Endocrinology and Metabolism, First Faculty of Medicine, Charles University and General University Hospital, Prague, Czech Republic; 5 Diabetes Centre, Institute for Clinical and Experimental Medicine, Prague, Czech Republic; 6 Department of Fetal Medicine, Gennet s.r.o., Prague, Czech Republic

**Keywords:** Type 1 diabetes mellitus, Serum, Plasma, Cord blood, Pregnancy complications

## Abstract

Pregnant women with type 1 diabetes mellitus (T1DM) are at higher risk of complication development in both mothers and their children. The present study aims to describe changes in circulating and umbilical cord concentrations of recently described predictors of pregnancy complications in a group of women with T1DM. Sixty-seven cases and 34 healthy pregnant controls were included in the study and circulatory levels of TGF-α, HB-EGF, BDNF, sFlt-4, PDGF, SCF, galectin-1, Fas ligand, CCL-20, P-selectin, IFNγR1, IL-10, IL-8, leptin, and insulin were assessed in 10 to 13, (V1), 18 to 21 (V2), 28 to 31 (V3) and 34 to 36 weeks of gestation (V4), and immediately after delivery (V5). BDNF, sFlt-4, HB-EGF, SCF, Fas ligand, galectin-1, IL-8, leptin, and insulin were higher in women with T1DM compared to controls during pregnancy (all p<0.05). While HB-EGF, CCL-20, and P-selectin correlate with maternal glucose control, circulatory SCF, P-selectin, galectin-1, PDGF, IFNγR1, sFlt-4, and TGF-α levels positively correlated with IL-10 levels suggesting that their expression is altered in the presence of inflammation. Leptin and insulin cord blood levels were higher in newborns of the mothers with T1DM relative to those without T1DM. Pregnancy of women with type 1 diabetes mellitus is associated with numerous changes in circulatory factors, but these changes are not reflected in the cord blood. The observed variations in trophic and inflammatory mediators may be linked to adverse pregnancy outcomes and could potentially be incorporated into predictive models for pregnancy complications in women with type 1 diabetes.

## Introduction

Type 1 diabetes mellitus (T1DM), also known as juvenile-onset diabetes or insulin-dependent diabetes mellitus, develops as a result of an autoimmune response targeted against pancreatic insulin-producing β cells resulting in insulin deficiency and hyperglycemia [1]. Pregnant women with T1DM are at higher risk of complication development for both mothers and their children, including preterm delivery, preeclampsia, macrosomia, shoulder dystocia, intrauterine fetal demise, fetal growth restriction, and congenital malformations [2]. Hyperglycemia is considered to be the trigger. Although the exact pathophysiological mechanisms are not fully understood, evidence suggests the involvement of inflammatory and metabolic mediators, such as TNF-α and leptin [3,4]. For this reason, we focused on novel predictors of preeclampsia and pregnancy complications and their possible associations with maternal T1DM. Furthermore, the effect of pregnancy on their circulatory levels, placental mRNA expression and cord blood levels are described and discussed.

**Fig. 1:**
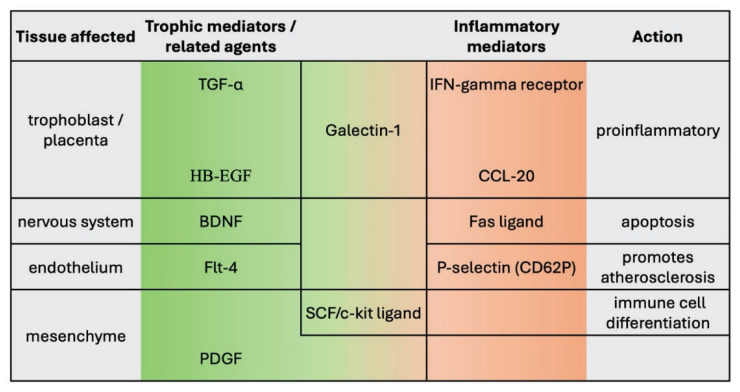
Effects of selected factors on the physiology of pregnancy.

For more clarity, the described factors were divided into two categories: trophic mediators and their related agents (i.e. TGF-α, HB-EGF, BDNF, sFlt-4, PDGF, SCF, Galectin-1) and cytokines/chemokines associated with inflammation and its regulation (Fas ligand, CCL-20, P-selectin, IFNγR1). The main functions are summarized in Figure 1.

**Transforming growth factor alpha (TGF-α)** is an epidermal growth factor family (EGF) member. TGF-α and TGF-β contribute to the physiological course of pregnancy. TGF-α is expressed in the human placenta and maternal-fetal interface cells throughout gestation and may act as an autocrine/paracrine regulator of trophoblast development and function [5,6]. **Heparin-binding epidermal growth factor-like growth factor (HB-EGF)**, another member of the EGF family, is involved in cellular proliferation, migration, adhesion, differentiation, growth, and development [7,8]. HB-EGF is important for implantation in which it works as an attachment and growth factor and for placenta development [9]. Its crucial role was first recognized given the high mortality rate seen in HB-EGF^−/−^ mice within the first postnatal week [10]. **Brain-derived neurotrophic factor (BDNF)** is the neurotrophic factor supporting the differentiation, maturation, and survival of neurons in the nervous system [11], where it stimulates EGF-induced proliferation and migration of human fetal stem/progenitor cells [12]. Besides that, it has a neuroprotective effect under hypoglycemia and plays a role in energy homeostasis, as shown by the BDNF administration resulting in suppression of energy intake and reduction of body weight [13,14]. Another less known predictor of pregnancy complication is **Flt-4** (also named as VEGFR3), a member of the VEGFR family and a relative to Flt-1 (VEGFR1), whose ratio with PlGF is widely used as a clinical predictor for impending preeclampsia and placenta related complications [15]. Flt-4 is present on all endothelial cells during the fetal development but becomes restricted to lymphatic endothelial cells and specific fenestrated vascular endothelial cells in adult life [16,17]. Also, **Platelet-derived growth factor (PDGF)** is structurally and functionally related to the VEGF family. PDGF is a potent growth factor for cells of mesenchymal origin. It is produced by several cell types, including placental and embryonic cells, in which it regulates oocyte growth and embryo development [18,19]. **SCF/c-kit ligand** is a pleiotropic glycoprotein with structural similarity of the VEGF family [20] triggering its biological effects by binding to the SCF receptor – tyrosine kinase encoded on the c-kit gene. Signal transduction is crucial in hematopoiesis, mast cell development, and the development of melanocytes and germ cells. Furthermore, SCF augments the effects of several hematopoietic growth factors produced in reproductive tissues during pregnancy and plays an essential role in cell migration, proliferation, and survival [21,22]. **Galectin-1** is a member of the galectin protein family capable of binding β-galactoside-rich glycoconjugates [23]. It is regulated by hormonal status, inflammatory mediators, and fluctuations in its redox status. It regulates feto-maternal tolerance as well as other physiological (placentation) and pathological processes (miscarriage, stillbirth, preeclampsia) during pregnancy [24,25]. Galectin-1 induces vascular permeability through the Flt-1 regulation [26] and is implicated in modulating cell-cell and cell-matrix interactions. It may also act as an autocrine negative growth factor that regulates cell proliferation. Besides, galectin-1 acts as an important inflammatory regulator due to its ability to act as an anti-inflammatory as well as pro-inflammatory regulator of monocytes, macrophages, or T lymphocytes [27].

Furthermore, we also focused on selected inflammatory mediators potentially connected with pregnancy complications.

The Fas pathway is the classic external pathway for cell death. The **Fas ligand** is a cell surface molecule belonging to the tumor necrosis factor family predominantly expressed on activated T and NK cells or present in trimeric soluble form. The Fas ligand initiates apoptosis by binding to the Fas receptor on the target cell triggering cell death through activation of caspase 8 [28,29]. Fas/Fas ligand pathway is potentially responsible for β-cell destruction in T1DM. Thus, it was suggested as a potential therapeutic target [30,31]. **CCL-20** is Th17-associated chemokine. Its production is induced by IL-17 [32]. Elevated levels of CCL-20 were reported in both T2DM and obesity [33,34] and related to pro-inflammatory reactions. **P-selectin (CD62P)** is an adhesion molecule expressed on activated platelets and endothelium, which is shed into circulation. It mediates the rolling of monocytes on activated endothelium, induces procoagulant microparticle formation, and promotes atherosclerotic development [35,36].

**IFN-gamma receptor** is comprised of two ligand-binding IFNγR1 chains associated with two signal-transducing IFNγR2 chains, belonging to the class II cytokine receptor family [37]. IFNγR1 polymorphisms contribute to preeclampsia pathophysiology [38] and the amount of IFNγR1^+^ monocytes and the intensity of IFNγR1-expression were increased in preeclampsia [39].

To complete the clinical picture, we assessed levels of **IL-8, IL-10, insulin**, and **leptin** and correlated them with all selected factors. In the case of IL-6 and TNF-α, analysis was insufficient for most samples, and therefore the data are not presented. IL-8 is a pro-inflammatory cytokine attracting and activating neutrophils [40], while IL-10 is associated with anti-inflammatory reactions and is produced by T cells, macrophages, dendritic cells, eosinophils and mast cells [41].

In this study, we aimed to determine circulatory levels of the afore-mentioned factors in women with T1DM and healthy controls to evaluate the effect of T1DM. At the same time, we determined their concentrations in the cord blood of the newborns and mRNA expression in the placenta.

## Methods

### Study subjects

Sixty-seven pregnant women with T1DM and 34 pregnant women without T1DM were included in the study. Women with no history of diabetes and normal glucose tolerance in pregnancy comprised the control group. All women in the study were recruited between 10 and 13 weeks of gestation. Among them, 43 % of women with type 1 diabetes mellitus (T1DM) planned their pregnancies and attended preconception care. The exclusion criteria included fetal congenital malformations and multiple pregnancies. Written informed consent was signed by each subject and the studies were approved by the Human Ethics Review Board, First Faculty of Medicine and General University Hospital, Prague, Czech Republic.

### Blood and tissue sampling

In pregnant women, a clinical examination was performed and blood samples were taken at 10 to 13 (visit 1 – V1), 18 to 21 (visit 2 – V2), 28 to 31 (visit 3 – V3), and 34 to 36 (visit 4 – V4) weeks of gestation. Blood samples were taken after overnight fasting and centrifuged for 10 min at 3000× g within 30 min after withdrawal. Serum or plasma aliquots were subsequently stored at −80 °C.

Placental biopsy, umbilical cord, and maternal blood samples for research were taken during labor (visit 5 – V5). Placental samples were immediately placed in the RNAlater® solution (Ambion® – Invitrogen, Carlsbad, California, USA) and were stored at −80 °C. Maternal and umbilical cord blood was centrifuged for 10 min at 3000× g within 30 min after withdrawal. Serum or plasma aliquots were subsequently stored at −80 °C.

All samples were taken at the Clinic of Gynecology, Obstetrics and Neonatology, First Faculty of Medicine and General University Hospital, Prague, Czech Republic.

### Hormonal and biochemical assays

Magnetic bead-based multiplex assay for the Luminex® platform (R&D Systems, Inc., McKinley Place NE, Minneapolis, USA) was used for detection of circulatory levels of TGF-α (sensitivity 0.820 pg/ml), HB-EGF (sensitivity 0.4 pg/ml), BDNF (sensitivity 0.320 pg/ml), VEGFR3/sFlt-4 (sensitivity 2.7 pg/ml), PDGF (sensitivity 0.200 pg/ml), SCF/c-kit ligand (sensitivity 1.4 pg/ml), galectin-1 (sensitivity 632 pg/ml), Fas Ligand/TNSF6 (sensitivity 1.2 pg/ml), CCL-20 (sensitivity 3.4 pg/ml), P-selectin/CD62P (sensitivity 0.9 pg/ml), IFNγR1/CD119 (sensitivity 0.1 pg/ml), IL-8/CXCL8 (sensitivity 1.8 pg/ml), IL-10 (sensitivity 1.6 pg/ml) and insulin (sensitivity 9.91 pg/ml). ELISA assay was used for leptin detection (Biovendor, Brno, Czech Republic, sensitivity 0.2 ng/ml). The intra- and interassay variabilities for all assays were between 5.0 and 10.0 %.

Biochemical parameters (urea, creatinine, uric acid, total bilirubin, alanine aminotransferase (ALT), aspartate aminotransferase (AST), γ-glutamyltransferase (GGT), glycated hemoglobin – HbA1c, glycemia, free thyroxine (fT4), thyroid stimulating hormone (TSH), erythrocytes, hematocrit, hemoglobin, leukocytes, and thrombocytes) were measured at the Institute of Medical Biochemistry and Laboratory Diagnostics, First Faculty of Medicine and General University Hospital, Prague, Czech Republic by standard laboratory methods.

### Quantitative real-time PCR

Samples of placenta were homogenized on MagNA Lyser Instrument (Roche Diagnostics GmbH, Mannheim, Germany). Total RNA was extracted on the MagCore Plus II instrument (RBC Bioscience Corp, New Taipei City, Tchaj-wan). RNA concentration was determined from the absorbance at 260 nm on NanoDrop One (Thermo Science/Thermo Fisher Scientific Massachusetts, USA). Reverse transcription was performed using random primers according to the manufacturer’s protocol of the High-Capacity cDNA Reverse Transcription Kits (Applied Biosystems, Foster City, CA, USA). Reverse transcription was performed using 0.25 μg of total RNA to synthesize the first-strand cDNA. Gene expression was performed on a ViiATM 7 Real-time PCR system (Applied Biosystems, Foster City, CA, USA). For reaction, a mix of TaqMan® Universal PCR Master Mix II, NO AmpErase® UNG (Applied Biosystems, Foster City, CA, USA), nuclease-free water (Fermentas Life Science, Vilnius, Lithuania) and specific TaqMan® Gene Expression Assays (Applied Biosystems, Foster City, CA, USA) were used. Beta 2 microglobulin was applied as an endogenous reference. The formula 2^−ddCt^ was used to calculate relative gene expression.

### Statistical analysis

Statistical analysis was performed and graphs were drawn using SigmaPlot 13.0 (SPSS Inc., Chicago, IL, USA). Results are expressed as means ± standard error of the mean (SEM). One-way ANOVA/One-way RM ANOVA followed by Holm-Sidak test, One-way ANOVA on Ranks/One-way RM ANOVA on Ranks followed by Dunn’s method, unpaired *t*-test or Mann-Whitney Rank Sum Test, were used for the assessment of intergroup differences, as appropriate. Pearson or Spearman correlation test was used to assess the association between biochemical parameters and measured maternal factors. Multiple linear regression using the Backward Stepwise method was used to assess the association between cord blood levels of assessed factors (dependent variables) and delivery outcomes (preeclampsia, birthweight, umbilical artery pH, neonatal jaundice, neonatal hypoglycemia, and cord blood levels of leptin, insulin, IL-10, and IL-8 were included in the model as independent variables). Results were adjusted for infant sex. Statistical significance was assigned to p<0.05.

## Results

### The effect of pregnancy and type 1 diabetes mellitus: the clinical picture

The maternal age in both groups was comparable (Table 1). The BMI before and during pregnancy was higher in women with T1DM compared to controls, and the prevalence of thyreopathy was also higher. As expected, pregnant women with T1DM had higher urea and glycated hemoglobin during pregnancy. Furthermore, at V2, higher erythrocyte count, hematocrit and hemoglobin levels were observed (Table 2).

An increase in insulin consumption was observed from the third trimester (V3). Mean glycemia decreased after the first trimester (V1) and the standard deviation gradually decreased during pregnancy which results in decreased glucose variability. The percentage of TIR (time in range; the percentage of time spent with CGM glucose levels in target 3.5–7.8 mmol/l) increased steadily during pregnancy, while the percentage of TBR (time below range; the percentage of time spent with CGM glucose levels below target 3.5 mmol/l) and TAR (time above range; the percentage of time spent with CGM glucose levels above target 7.8 mmol/l) both decreased following the first trimester (V1) (Table 3).

### The effect of pregnancy and type 1 diabetes mellitus on systemic levels of selected parameters

**Fig. 2:**
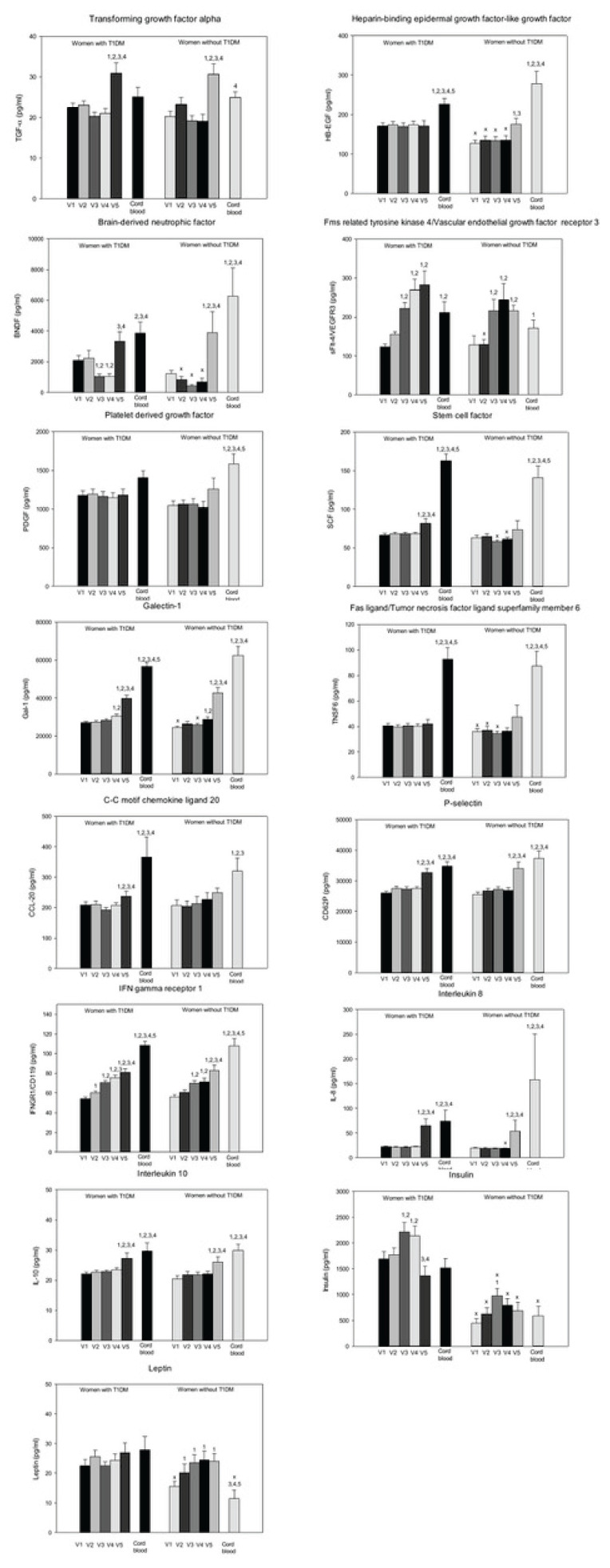
Circulatory and cord blood levels of selected parameters in pregnant women with and without type 1 diabetes mellitus. V1: 10 to 13 weeks of gestation. V2: 18 to 21 weeks of gestation. V3: 28 to 31 weeks of gestation. V4: 34 to 36 weeks of gestation. V5: delivery. Values are Mean ± SEM. ^1^ p<0.05 vs. V1; One Way repeated measures ANOVA/One Way repeated measures ANOVA on Ranks. ^2^ p<0.05 vs. V2; One Way repeated measures ANOVA/One Way repeated measures ANOVA on Ranks. ^x^ p<0.05 vs. T1DM; unpaired *t*-test or Mann-Whitney Rank Sum Test. In case of cord blood: ^1^ p<0.05 vs. maternal serum levels on V1; ^2^ p<0.05 vs. maternal serum levels on V2; ^3^ p<0.05 vs. maternal serum levels on V3. All One Way ANOVA/One Way ANOVA on Ranks.

TGF-α concentrations at delivery (V5) were higher than during pregnancy regardless of T1DM status. Women with T1DM had higher HB-EGF concentrations throughout pregnancy (V1–4) but comparable concentrations at delivery. BDNF concentrations were also elevated from the second trimester (V2–V4) in women with T1DM despite a significant decrease at V3 and V4 and become highest at delivery. In both groups, the concentrations of sFlt-4 increased from the third trimester to delivery (V3–5). No differences were observed between groups except for higher concentration in the second trimester in women with T1DM. Neither pregnancy nor T1DM affected PDGF concentrations. SCF concentrations were stable in both groups during pregnancy, but there was an increase in women with T1DM at delivery. In addition, SCF concentrations were elevated in the third trimester (V3–4) compared to healthy controls. Galectin-1 concentration exhibited comparable trends in both groups. It was stable until the late third trimester (V4) and peaked at delivery (V5). Women with T1DM had higher concentrations at V1 and V3. In both groups, the Fas ligand concentrations were stable during pregnancy and delivery, with higher concentrations observed in women with T1DM before the late third trimester (V1–3). The concentrations of CCL-20 were also stable during pregnancy, with no differences observed between the groups. In both groups, increased P-selectin was observed at delivery (V5) compared to pregnancy, but T1DM status did not affect its concentration. In both groups, the IFNγR1 increased gradually during pregnancy and delivery (V1–5) and the concentrations were comparable between the groups. IL-8 and IL-10 increased at delivery (V5) in both groups. Women with T1DM exhibited higher concentrations of IL-8 in the late third trimester (V4). At each study visit, insulin concentrations were higher in women with T1DM. In women with T1DM, the concentrations peaked in the third trimester (V3–4) and dropped at delivery (V5). While leptin levels were unchanged in women with T1DM, healthy controls had lower concentrations in the first trimester (V1) compared to the latter trimesters and women with T1DM. The results are summarized in Figure 2.

### The effect of pregnancy and type 1 diabetes mellitus on delivery outcomes, mRNA expression in the placenta, and cord blood levels of selected parameters

The birthweight of newborns was higher in women with T1DM despite a lower gestational age at delivery. Furthermore, these women experienced hypertensive disorders in pregnancy and underwent cesarean sections more often. No cases of fetal growth restriction or HELLP syndrome were observed in the studied population. Additionally, newborns of T1DM mothers had a higher incidence of neonatal hypoglycemia (Table 4).

Placental mRNA expressions of IL-10 and TNF-α were higher in women with T1DM compared to healthy controls (Table 5). Of all the studied factors, only leptin and insulin concentrations were higher in the cord blood of women with T1DM (Fig. 2). The associations of circulatory factors in cord blood with clinical outcomes are present in Table 6.

In the majority of circulatory factors, with the exception of TGF-α and leptin in T1DM and insulin in both groups, significant differences were observed between umbilical cord and maternal pregnancy concentrations. Cord blood HB-EGF, SCF, Fas ligand, galectin-1, PDGF and IFNγR1 concentrations were higher compared to maternal levels at the time of delivery (Fig. 2).

## Discussion

The goal of this study was to determine the effects of long-lasting type 1 diabetes mellitus with good diabetes control on circulatory levels of trophic and inflammatory factors associating with pregnancy complications such as preeclampsia, preterm delivery, macrosomia and others.

**TGF-α** was described as a trophic factor involved in physiological placentation [6] and fetal growth. Increased TGF-α cord blood levels were previously reported in fetuses with fetal growth restriction [42]. In line, we observed an association of its cord blood levels with preeclampsia and birthweight. Both maternal and fetal TGF-α levels correlated with IL-10. Nevertheless, comparable pregnancy concentrations of TGF-α with an increase at the time of delivery were observed in both groups. Since maternal and umbilical cord TGF-α concentrations were not affected by glucose control or diabetes treatment, we consider the effect of T1DM to be negligible. In contrast, another member of the EGF family **HB-EGF** exhibited increased levels in women with T1DM during the whole pregnancy, and maternal concentrations positively correlated with insulin levels (r=0.356). Regardless of T1DM, HB-EGF cord blood levels were higher compared to maternal levels and were associated with birthweight. Given the association with insulin levels and published results in the pediatric population [43], it is affected by T1DM. Transactivation of the epidermal growth factor receptor in mesangial cells and HB-EGF contributes to glomerular matrix accumulation, leading to diabetic kidney disease [44]. Furthermore, it is actively involved in the development of other metabolic complications of diabetes in the liver and cardiovascular system [45]. HB-EGF is one of the first mediators involved in trophoblast implantation. It plays a key role in the cytoprotection of the developing placenta under conditions of hypoxia and oxidative stress [9]. Thus, the observed elevated HB-EGF concentrations in pregnancy may mirror efforts to slow placental senescence in the presence of T1DM. Another growth factor related to TGF signaling, **BDNF**, was increased in women with T1DM compared to controls between 18 and 36 weeks of gestation. Our results are consistent with previously published data [47], but on the contrary, in one study, women with T1DM had lower baseline BDNF levels compared to healthy controls [48]. The different conclusions can probably be explained by the differences in diabetes duration before pregnancy and baseline insulin resistance, as longer duration and higher insulin resistance are associated with diminished concentration of neurotrophins [46]. Consistent with another study [47], we observed a drop in BDNF concentration in the third trimester in women with T1DM, which could be explained by the physiologic increase of insulin resistance in the second half of the pregnancy. We could not demonstrate a correlation of BDNF concentration with other factors and markers. One animal study showed that dietary restrictions are associated with increased BDNF in the brain and decreased concentrations of glucose, insulin, and leptin in obese diabetic mice [48]. BDNF cord blood levels were insignificantly lower in the non-T1DM group (p=0.135), similar to the previously reported decrease in GDM [49]. Although both groups umbilical cord concentrations were higher than maternal pregnancy concentrations, they were comparable with maternal concentrations during delivery. This contrasts with another study [52] in which the umbilical cord concentration was lower. The authors hypothesize that BDNF, like other neuroendocrine factors, plays a role in triggering the delivery. This may explain why we did not observe the same decrease in BDNF concentrations, as a significant number of deliveries were carried out by elective cesarean sections prior to the labor onset.

The concentration of **sFlt-4** during pregnancy and at birth did not differ significantly in the groups studied and showed a similar trend, except for a higher concentration in diabetic women between 18 and 21 weeks. sFlt-4 levels were associated with maternal and fetal IL-10 levels. Its placental mRNA expression did not differ between the groups. Relevant literature data regarding sFlt-4 and its role in physiological and pathological pregnancy complicated by diabetes are not available.

In contrast, **PDGF** levels were constant during pregnancy, and placental expression was not influenced by T1DM, which contradicts the findings of a smaller earlier study [50]. Authors assume that a lower concentration of PDGF could be related to enhanced cell proliferation in the settings of diabetic microangiopathy. PDGF levels positively correlated with maternal IL-10 levels and leukocyte and platelet counts (all r<0.335), suggesting a link to the inflammatory process rather than diabetes itself.

In women with T1DM, **Galectin-1** levels were higher up to the early third trimester compared to controls. Studies focusing on patients with T2DM showed an inverse association with glucose levels [51]. We found its positive association with BMI, glycated hemoglobin, leptin, insulin, IL-8 and IL-10 levels supporting a galectin-1 interconnection with glucose control, insulin resistance and inflammation. Previous studies confirmed its regulation through hormonal and inflammatory mediators [24,25]. At the time of delivery, galectin-1 levels peak in both women with T1DM and healthy controls but not in women with gestational diabetes as was previously described [52]. Cord blood Galectin-1 concentrations were higher compared to maternal levels and correlated with IL-10 and leptin cord blood levels.

Similarly to factors with trophic properties, cytokines, and chemokines do not exhibit distinct differences in association with T1DM. We observed higher concentrations of **Fas ligand** up to 31 weeks in women with T1DM, similar to what was previously demonstrated in smoking or preeclamptic women [53,54]. These results may reflect increased susceptibility to pregnancy complications in women with T1DM. Fas ligand expression in trophoblast cells is involved in maternal immune tolerance of allogenic fetus through the regulation of apoptosis in activated lymphocytes. Thus Fas ligand plays an important role in the maintenance of pregnancy, normal fetal development [55,56], but also in the pathophysiology of preeclampsia [54,57]. An increased Fas ligand in placental-derived extracellular vesicles of women with preeclampsia. Moreover, maternal Fas ligand concentrations in T1DM pregnancies correlated with glycated hemoglobin (r=0.287). In both groups, Fas ligand levels were stable and cord blood levels were higher than maternal levels in both groups, as was also described previously [58]. In the case of **CCL-20**, we found no difference between T1DM and control groups throughout the pregnancy and delivery; in women with T1DM, CCL-20 increased during delivery compared to maternal pregnancy concentrations. It was one of the few factors to show an association with glycemia and the percentage of daily time spent in hyperglycemia. As CCL-20 is associated with pro-inflammatory Th17 cell response, our data suggest glucose control in pregnancy directly affects maternal inflammatory response. Furthermore, CCL-20 cord blood levels were associated with preeclampsia and fetal leptin, insulin, IL-8, and IL-10 levels, suggesting its role in pregnancy complications, fetal adiposity, and inflammation. The course of maternal **P-selectin** concentrations during pregnancy, at birth and cord blood concentrations was identical in both groups of women studied. We observed an increase in maternal concentrations at delivery, which were comparable to the umbilical cord concentrations. Maternal P-selectin concentrations in women with T1DM correlated with glucose control, positively with TAR (r=0.262) and negatively with TIR (r=−0.287). Increased concentrations of P-selectin were previously described in pregnant women with T1DM with diabetic microangiopathy [59], preeclampsia, and fetal growth restriction [60]. **IFNγR1** is a part of IFN-γ cytokine receptor triggering a pro-inflammatory signaling cascade [37]. IFNγR1 levels gradually increased throughout the pregnancy, and T1DM had a negligible effect. Furthermore, IFNγR1 was not associated with parameters of glucose control, but only with inflammatory markers. This was further confirmed in the cord blood by its association with IL-10 cord blood levels. IFNγR1 cord blood levels were higher compared to maternal levels at the time of delivery.

To sum up, type 1 diabetes mellitus in pregnancy is associated with numerous changes in maternal circulating factors involved in fetal growth and inflammation, including HB-EGF, BDNF, Galectin-1 and Fas ligand, which may contribute to adverse pregnancy outcomes. We found that HB-EGF, CCL-20, and P-selectin correlate with maternal glucose control. On the other hand, circulatory SCF, P-selectin, galectin-1, PDGF, IFNγR1, sFlt-4 and TGF-α levels positively correlated with IL-10 levels suggesting that their expression is altered in the presence of inflammation. Importantly, changes in maternal concentrations of the studied parameters did not translate into cord blood concentrations, with the exception of leptin and insulin which were higher in children of mothers with T1DM.

## Figures and Tables

**Table 1:** The characteristics of the participating women.

	Pregnant women with T1DM (V1)	Pregnant women without T1DM (V1)
*Number (n)*	67	34
*Age (year)*	31.50±0.52	32.34±0.82
*BMI (kg/m* *^2^* *)*	25.18±0.59	21.45±0.36^x^
*Nicotinism*	4 (5.97 %)	1 (2.94 %)
*Thyreopathy*	29 (43.28 %)	6 (17.65 %)^x^
*Essential hypertension*	3 (4.48 %)	1 (2.94 %)
*Duration of T1DM (year)*	14.60±0.87	---
*Diabetic kidney disease*	4 (5.97 %)	---
*Diabetic rethinopathy*	13 (19.40 %)	---
*Diabetic neuropathy*	1 (1.49 %)	---
*Insulin pump*	37 (55.22 %)	---
*HbA1c (mmol/l)*	51.59±1.97	---

Values are Mean ± SEM or count (percentage).

x p<0.05 vs. pregnant women with T1DM; unpaired *t*-test or Mann-Whitney Rank Sum Test.

**Table 2:** Biochemical parameters of study subjects during pregnancy.

	Pregnant women with T1DM				Pregnant women without T1DM			
	V1	V2	V3	V4	V1	V2	V3	V4
*Gestation age (week)*	12.4±0.19	20.8±0.13	30.23±0.17	34.49±0.12	12.77±0.19	20.71±0.16	30.25±0.24	35.00±0.27
*BMI (kg/m* *2* *)*	25.94±0.57	27.48±0.59^1^	28.82±0.52^1^,^2^	29.71±0.54^1^,^2^,^3^	21.43±0.40^x^	22.73±0.37^1^,^x^	24.67±0.42^1^,^2^,^x^	25.45±0.57^1^,^2^,^3^,^x^
*HbA1c (mmol/mol)*	44.16±1.22	38.21±0.84^1^	40.36±0.80^1^,^2^	40.90±0.87^1^,^2^	31.55±0.63^x^	27.97±0.60^1^,^x^	30.80±0.58^2^,^x^	32.05±0.67^2^,^x^
*Creatinine (μmol/l)*	53.61±1.22	54.89±3.39	52.28±1.29	55.63±1.70	50.08±1.96	49.10±1.10	51.31±1.54	51.27±1.85
*Uric acid (mmol/l)*	180.4±5.3	191.7±6.1	211.1±6.4^1^,^2^	255.1±8.1^1^,^2^,^3^	175.0±5.6	192.7±4.5^1^	209.4±6.8^1^,^2^	226.4±12.1^1^,^2^,^3^
*Urea (mmol/l)*	3.423±0.123	3.348±0.122	2.997±0.122^1^,^2^	3.233±0.151	2.980±0.107^x^	2.957±0.139^x^	2.781±0.090	2.645±0.135^x^
*Total bilirubin (μmol/l)*	6.257±0.482	5.492±0.373	4.873±0.313^1^	5.720±0.441	6.193±0.85	7.086±1.934	4.820±0.502	8.348±2.695
*ALT (μkat/l)*	0.384±0.130	0.297±0.018	0.32±0.020	0.334±0.030	0.244±0.013	0.322±0.042	0.297±0.033	0.287±0.038
*AST (μkat/l)*	0.311±0.008	0.334±0.011	0.348±0.013^1^	0.401±0.019^1^,^2^,^3^	0.318±0.009	0.357±0.0186	0.369±0.023	0.370±0.037
*GGT (μkat/l)*	0.209±0.020	0.158±0.010^1^	0.150±0.008^1^	0.163±0.015^1^	0.193±0.022	0.143±0.0103	0.346±0.162	0.363±0.146
*fT4 (pmol/l)*	15.21±0.43	14.10±0.34^1^	14.00±0.24^1^	13.77±0.28^1^	12.45±0.90	12.18±0.71^x^	11.20±0.83^x^	12.82±0.31^x^
*TSH (pmol/l)*	1.962±0.167	2.242±0.286	1.903±0.138	2.187±0.166	2.785±0.733	2.827±0.624	3.358±0.782	1.965±0.174
*Erythrocytes (10* ^*^ *12/l)*	4.313±0.055	3.976±0.040^1^	3.881±0.042^1^	3.983±0.040^1^	4.157±0.050	3.765±0.047^1^,^x^	3.800±0.053^1^	3.858±0.049^1^
*Hematocrit (%)*	37.77±0.25	35.82±0.32^1^	34.93±0.35^1^,^2^	35.70±0.34^1^,^3^	47.54±10.52	34.58±0.34^x^	34.50±0.47	34.64±0.48
*Hemoglobin (g/l)*	127.5±1.0	120.7±1.1	117.2±1.3^1^,^2^	117.5±1.3^1^,^3^	125.0±1.4	116.0±1.4^1^,^x^	115.2±1.8^1^	114.7±1.8^1^
*Leukocytes (10* ^*^ *9/l)*	8.660±0.197	9.753±0.299^1^	9.591 ± 0.298 1	9.204±0.283	8.409±0.339	9.122±0.398	9.198±0.431	9.253±0.506
*Thrombocytes (10* ^*^ *9/l)*	266.6±7.3	262.3±7.9	243.5±8.7	225.7±7.6	232.2±7.0	212.4±10.4	223.9±8.2	199.4±11.7

V1: 10 to 13 weeks of gestation. V2: 18 to 21 weeks of gestation. V3: 28 to 31 weeks of gestation. V4: 34 to 36 weeks of gestation. ALT – alanine aminotransferase. AST – aspartate aminotransferase. GGT – γ-glutamyltransferase. Values are Mean ± SEM.

1 p<0.05 vs. V1; one-way repeated measures ANOVA/one-way repeated measures ANOVA on Ranks.

2 p<0.05 vs. V2; one-way repeated measures ANOVA/one-way repeated measures ANOVA on Ranks.

x p<0.05 vs. pregnant women with T1DM; unpaired *t*-test or Mann-Whitney Rank Sum Test.

**Table 3:** Glucose control in women with T1DM during pregnancy.

	Pregnant women with T1DM			

	V1	V2	V3	V4
*Insulin dosage*	38.454±1.722	40.484±1.730	55.914±2.283^1^,^2^	64.407±2.836^1^,^2^,^3^
*HbA1c (mmol/l)*	44.16±1.22	38.21±0.84^1^	40.36±0.80^1^,^2^	40.90±0.87^1^,^2^
*Average glycemia from sensor (mmol/l)*	6.958±0.192	6.475±0.116^1^	6.521±0.105^1^	6.375±0.097^1^
*Average glycemia from sensor – standard deviation (mmol/l)*	2.307±0.086	2.130±0.083^1^	2.021±0.071^1^,^2^	1.857±0.062^1^,^2^,^3^
*TBR (%)*	4.9±0.6	5.5±0.7	4.7±0.6	4.3±0.7^1^,^2^
*TIR (%)*	66.1±2.3	71.6±1.7^1^	71.7±1.6^1^	75.8±1.5^1^,^2^,^3^
*TAR (%)*	29.1±2.4	24.0±1.9^1^	23.7±1.8^1^	20.1±1.5^1^,^2^

V1: 10 to 13 weeks of gestation. V2: 18 to 21 weeks of gestation. V3: 28 to 31 weeks of gestation. V4: 34 to 36 weeks of gestation. Values are Mean ± SEM.

1 p<0.05 vs. V1; one-way repeated measures ANOVA/one-way repeated measures ANOVA on Ranks.

2 p<0.05 vs. V2; one-way repeated measures ANOVA/One-way repeated measures ANOVA on Ranks.

TIR (time in range) the percentage of time spent with CGM glucose levels in target 3.5–7.8 mmol/l, TBR (time below range) the percentage of time spent with CGM glucose levels below target 3.5 mmol/l, TAR (time above range) the percentage of time spent with CGM glucose levels above target 7.8 mmol/l.

**Table 4:** Perinatal outcomes.

	Pregnant women with T1DM	Pregnant women without T1DM
*Gestation age at delivery (week)*	37.6 ± 0.3	38.5 ± 0.7^x^
*Cesarean section*	42 (66.67 %)	9 (32.14%)^x^
*Vacuum extraction*	2 (3.17 %)	0 (0 %)
*Pregnancy induced hypertension and preeclampsia*	8 (11.27 %)	0 (0 %)^x^
*Intrahepatic cholestasis of pregnancy*	3 (4.76 %)	0 (0 %)
*EFW≥4000 g*	17 (26.98 %)	4 (13.79 %)
*Birthweight (g)*	3631.1 ± 84.8	3268.3 ± 140.4^x^
*Newborn males*	29 (47.54 %)	13 (44.83 %)
*Newborn females*	32 (52.46 %)	16 (55.17 %)
*Neonatal jaundice*	18 (28.57 %)	5 (17.24 %)
*Neonatal hypoglycemia*	29 (46.03 %)	1 (3.45 %)^x^
*Respiratory distress syndrome*	4 (6.35 %)	0 (0 %)

Values are Mean ± SEM or count (percentage).

x p<0.05 vs. pregnant women with T1DM; unpaired *t*-test or Mann-Whitney Rank Sum Test.

**Table 5:** mRNA expression of selected parameters in the placenta.

	Women with T1DM	Women without T1DM
*β-HCG*	3.308 ± 0.765	1.716 ± 0.665
*PAPP-α*	1.486 ± 0.191	1.746 ± 0.413
*PP13*	1.825 ± 0.262	1.662 ± 0.389
*Flt-4*	1.326 ± 0.176	1.562 ± 0.385
*PDGF*	1.746 ± 0.198	1.122 ± 0.132
*TGF-β*	1.054 ± 0.073	1.092 ± 0.127
*IL-10*	1.546 ± 0.114	1.083 ± 0.141^x^
*IL-6*	1.722 ± 0.207	1.184 ± 0.188
*IL-8*	4.666 ± 2.295	1.225 ± 0.263
*MCP-1*	1.345 ± 0.115	1.123 ± 0.155
*TNF-α*	1.606 ± 0.097	1.078 ± 0.110^x^
*IFN-γ*	1.684 ± 0.324	1.417 ± 0.375

Values are Mean ± SEM.

x p<0.05 vs. pregnant women with T1DM; unpaired *t*-test or Mann-Whitney Rank Sum Test.

**Table 6:** Associations of circulatory factors in cord blood with clinical outcomes.

	r^2^	Preeclampsia	Birthweight	Neonatal jaundice	Leptin	Insulin	IL-10	IL-8
*TGF-α*	0.441	0.003	0.043				0.035	
*HB-EGF*	0.197		0.006					
*BDNF*	0.147		0.014					
*sFlt-4*	0.087						0.045	
*PDGF*	0.150			0.004				
*SCF*	0.192						0.004	
*Galectin-1*	0.441				0.038		<0.001	
*Fas ligand*	0.089						0.043	
*CCL-20*	0.171				0.017			
*P-selectin*	0.621	<0.001			0.047	0.002	<0.001	0.047
*IFNγR1*	0.089						0.043	

Significant p-values (p<0.05) are placed in the table for single parameters.

## Abbreviations

ALT: alanine aminotransferase
ANOVA/RM ANOVA: analysis of variance/repeat measurement analysis of variance
AST: aspartate aminotransferase
BDNF: brain-derived neurotrophic factor
BMI: Body Mass Index
CCL: CC chemokine ligands
CGM: continuous glucose monitor
CRP: C-reactive protein
fT4: free thyroxine
GGT: γ-glutamyltransferase
HbA1c: Hemoglobin A_1c_/glycated hemoglobin
HB-EGF: heparin-binding epidermal growth factor-like growth factor
IFNγR1: interferon gamma receptor
IL: interleukin
mRNA: messenger ribonucleic acid
PCR: polymerase chain reaction
PDGF: platelet-derived growth factor
CF: c-kit ligand
SEM: standard error of the mean
sFlt: soluble fms-like tyrosine kinase
TAR: time above range
TBR: time below range
T1DM: type 1 diabetes mellitus
TGF-α/β: transforming growth factor alpha/beta
TIR: time in range
TNF: tumor necrosis factor
TSH: thyroid stimulating hormone
VEGFR: receptor for vascular endothelial growth factor
